# Phase-specific differential regulation of mechanical allodynia in a murine model of neuropathic pain by progesterone

**DOI:** 10.3389/fphar.2023.1253901

**Published:** 2023-12-11

**Authors:** Sheu-Ran Choi, Dae-Hyun Roh, Ji-Young Moon, Alvin J. Beitz, Jang-Hern Lee

**Affiliations:** ^1^ Department of Pharmacology, Catholic Kwandong University College of Medicine, Gangneung, Republic of Korea; ^2^ Department of Veterinary Physiology, College of Veterinary Medicine and Research Institute for Veterinary Science, Seoul National University, Seoul, Republic of Korea; ^3^ Department of Oral Physiology, College of Dentistry, Kyung Hee University, Seoul, Republic of Korea; ^4^ Animal and Plant Quarantine Agency, Gimcheon, Republic of Korea; ^5^ Department of Veterinary and Biomedical Sciences, College of Veterinary Medicine, University of Minnesota, St Paul, MN, United States

**Keywords:** progesterone, cytochrome P450c17, 5α-reductase, astrocytes, mechanical allodynia, sciatic nerve injury

## Abstract

Progesterone has been shown to have neuroprotective capabilities against a wide range of nervous system injuries, however there are negative clinical studies that have failed to demonstrate positive effects of progesterone therapy. Specifically, we looked into whether progesterone receptors or its metabolizing enzymes, cytochrome P450c17 and 5α-reductase, are involved in the effects of progesterone on neuropathic pain after chronic constriction injury (CCI) of the sciatic nerve in mice. Intrathecal progesterone administration during the induction phase of chronic pain enhanced mechanical allodynia development and spinal glial fibrillary acidic protein (GFAP) expression, and this enhancement was inhibited by administration of ketoconazole, a P450c17 inhibitor, but not finasteride, a 5α-reductase inhibitor. Furthermore, phospho-serine levels of P450c17 in the spinal cord were elevated on day 1 after CCI operation, but not on day 17. In contrast, intrathecal progesterone administration during the maintenance phase of chronic pain decreased the acquired pain and elevated GFAP expression; this inhibition was restored by finasteride administration, but not by ketoconazole. The modification of mechanical allodynia brought on by progesterone in CCI mice was unaffected by the administration of mifepristone, a progesterone receptor antagonist. Collectively, these findings imply that progesterone suppresses spinal astrocyte activation via 5α-reductase activity during the maintenance phase of chronic pain and has an analgesic impact on the mechanical allodynia associated with the growing neuropathy. Progesterone, however, stimulates spinal astrocytes during the induction stage of peripheral neuropathy and boosts the allodynic impact caused by CCI through early spinal P450c17 activation.

## 1 Introduction

The steroid hormones and metabolites generated by neurosteroidogenic enzymes in the central nervous system (CNS) are referred to as “neurosteroids” (Baulieu, 1997). Numerous investigations have demonstrated that neurosteroids exert powerful neuromodulatory effects in the neurological system (Baulieu, 1997; Haage and Johansson, 1999; Yoon et al., 2010). Progesterone, a neurosteroid, has been demonstrated to have neuroprotective effects in cell and animal models as well as in humans, most notably in experimental models of CNS injury (Arbo et al., 2016). Beyond its putative role in neuroprotection, recent studies have also suggested that progesterone has antinociceptive properties (Dableh and Henry, 2011; Jarahi et al., 2014). Progesterone and its reduced metabolite, allopregnanolone have been reported to alter nociceptive sensitivity and produce an analgesic effect in several rodent models of nerve injury (Charlet et al., 2008; Coronel et al., 2014; Jarahi et al., 2014; Patte-Mensah et al., 2014). Thus, the use of progesterone becomes a potential candidate for the development of new therapeutics in clinical trials, as well as, in experimental studies. However, on balance there is also a growing list of negative or inconclusive clinical trials, which have failed to show a beneficial effect of progesterone in the treatment of patients after acute CNS injury (Arbo et al., 2016). Because these discrepancies, it is critical to develop a better mechanistic understanding of the actions of progesterone on acute and chronic pain in order to develop novel steroid-based therapeutics, particularly to treat neuropathic pain.

Cytochrome P450c17 is an important neurosteroid-metabolizing enzyme, which can catalyze the conversion of progesterone into androstenedione via steroid 17α-hydroxylase and 17,20-lyase activities (Arbo et al., 2016). It has been suggested that androstenedione can be metabolized to testosterone and further converted to 17β-estradiol. In contrast to the allopregnanolone synthetic pathway initiated by 5α-reductase, this multistep process triggered by P450c17 from progesterone has been suggested to enhance allodynia in various nociceptive models (Tang et al., 2008; Smeester et al., 2016; Choi et al., 2018). Thus, P450c17 could play an important role as a branch point of progesterone metabolism that determines both the duration of actions of progesterone and the direction of nociceptive signal transmission. We have previously discovered that P450c17 expression is higher in spinal astrocytes after the sciatic nerve is injured (Choi et al., 2019c). We also found that blocking P450c17 in the early stages of pain reduces the development of the pain and the activity of astrocytes (Choi et al., 2019a; Choi et al., 2019c). Moreover, there is data suggesting that neurosteroids can modulate not only the morphology of astrocytes, but also the function of astrocytes and neurons in the CNS (Del Cerro et al., 1995; Arbo et al., 2016). Whether the activity of P450c17 changes in astrocytes of the lumbar spinal cord in accordance with pain remains to be determined, but it is interesting to speculate that an increase in astrocyte P450c17 activity could alter the effect of progesterone treatment on pain, as well as, on the pathophysiological changes that occur in astrocytes following peripheral neuropathy.

Thus, we examined whether: (1) intrathecal administration of progesterone affects mechanical allodynia and astrocyte activation in the spinal cord during the induction and maintenance phases of neuropathic pain in CCI mice; (2) sciatic nerve injury modulates the activity of spinal P450c17 via increase in the phospho-serine levels of P450c17; and (3) the P450c17 inhibitor, ketoconazole or the 5α-reductase inhibitor, finasteride attenuates the actions of progesterone on spinal astrocyte activation and mechanical allodynia induced by peripheral nerve injury.

## 2 Materials and methods

### 2.1 Animals and induction of neuropathic pain

We conducted experiments with male Crl:CD1(ICR) mice that were 5 weeks old (23 ± 2 g). Five-week-old mice were chosen because they show measurable nociceptive responses following nerve injury. To avoid the effect of female hormones on sensory perception, male mice were used in the present study. The animals were given a pellet diet and had access to water whenever they wanted. They lived in a place where there was light for 12 h and dark for 12 h each day. The temperature was controlled and maintained at 23°C ± 2°C. The experiments followed the rules set by the committees at Seoul National University (approval No. SNU-190219-2-2) and Catholic Kwandong University (approval No. CKU-2023-001 and CKU-2023-004).

Nerve chronic constriction injury (CCI) was done using a technique described by Bennett and Xie with a minor modification (Bennett and Xie, 1988). Under isoflurane anesthesia, the right sciatic nerve was exposed and three ligatures (6-0 silk) were loosely wrapped around the sciatic nerve under a neuro-surgical microscope. Sham surgery was carried by exposing the right sciatic nerve in the same way but without nerve ligation.

### 2.2 Drugs and intrathecal administration

Progesterone (4-Pregnene-3,20-dione; 10, 30, 100 nmol), mifepristone (a progesterone receptor antagonist; 0.1, 0.3, 1 nmol), ketoconazole (a P450c17 inhibitor; 1, 3, 10 nmol), and finasteride (a 5α-reductase inhibitor; 15, 50, 150 nmol) were obtained from Sigma–Aldrich (St. Louis, MO, United States). We selected the doses of all drugs based on the amounts used in previous studies (Takasaki et al., 2005; Choi et al., 2019c). All drugs were mixed with a solution containing 5% dimethyl sulfoxide in corn oil and given twice a day either during the early stage (from days 0 to 3 after surgery) or later stage (from days 14 to 17 after surgery) of neuropathic pain.

Intrathecal administration was performed to introduce drugs into the subarachnoid space of the spinal cord in the same way it was done before by Hylden and Wilcox (1980). Under isoflurane anesthesia, a 30-gauge needle connected to a 50 μL Hamilton syringe was used to put drugs between the L_5-6_ intervertebral space. The needle was successfully put into the intrathecal space based on the tail flick response. All intrathecal injections employed a 5 µL injection volume, while the control group received the same volume of vehicle (5% dimethyl sulfoxide in corn oil).

### 2.3 Behavioral assessment

We conducted the mechanical allodynia test in the same way it was done in a previous study (Choi et al., 2013). The % paw withdrawal response frequency (PWF) was measured by applying a von Frey filament, weighing 0.16 g, 10 times to the plantar surface of each injured hind paw. All animals were subjected to behavioral tests 1 day before surgery to establish appropriate baseline values for paw withdrawal reactions to mechanical stimulation. The animals were then divided into experimental and control groups at random. One group of mice (the induction group) were evaluated at 1, 2, 3, 6, and 9 days after surgery. Another group of mice (the maintenance group) were evaluated 14, 15, 16, 17, and 20 days after surgery. Behavioral tests were conducted by trained person at the same time of the day. All observations and analyses were done without knowing the details of the experimental conditions.

### 2.4 Co-immunoprecipitation and western blot assay

The phospho-serine levels of P450c17 at day 1 and 17 post-CCI surgery were detected by immunoprecipitation and Western blot assay as previously described (Choi et al., 2016). The spinal cord dorsal horn tissues were blended in a solution called IP Lysis/Wash Buffer. We used the BCA protein assay kit to estimate the amount of protein in samples. Then, we mixed the protein samples (a total of 300 µg of protein) with AminoLink Plus Coupling Resin. This substance was coupled with an antibody specific for P450c17 (a total of 10 μg, cat# ab125022, Abcam plc.). The proteins that were attached to the antibodies were separated using elution buffer and analyzed by Western blot assay using an anti-phosphoserine antibody (1:1,000, cat# ab6639, Abcam plc.) and an anti-P450c17 antibody (1:1,000, cat# ab125022, Abcam plc.). The particular bands were measured using ImageJ software (version 1.45s). The average value of the sham group was considered 100%. Then, the percentage change compared to the average value of the sham group was calculated in the CCI group.

### 2.5 Immunohistochemistry and image analysis

The process of immunohistochemistry was done at day 1 and 17 post-CCI surgery in the same way it was done in previous studies from our labs (Choi et al., 2016; Choi et al., 2019b). Under isoflurane anesthesia, perfusion was performed with calcium free tyrode’s solution and fixation was done by paraformaldehyde solution. We cut L_4-5_ spinal cords into sections that were 40 μm thick using a cryostat (Leica Biosystems, Germany). The sections of the spinal cord were put in a solution with a primary antibody specific for GFAP (1:1,000, cat# MAB360, Millipore Co.). Alexa Fluor^®^ 488-conjugated anti-mouse antibody (1:400, Life Technologies) was used as a secondary antibody. As a negative control, staining was performed without primary antibody. We used a confocal laser scanning (Nikon Eclipse TE2000-E) and a software (EZ-C1 Gold version 3.80, Nikon Instech Co., Ltd) to acquire fluorescent images.

To analyze the immunofluorescence of GFAP, we randomly chose tissue sections and used Metamorph computer program (version 7.7.2.0). This method has been used before and was described by Choi et al. (2019b) and Choi et al. (2016). Spinal cord dorsal horns were divided into three different regions, superficial dorsal horn (SDH, laminae I and II), nucleus proprius (NP, laminae III and IV), and neck region (NECK, laminae V and VI). The immunofluorescence positive pixels were counted by measuring the percentage of the pixel area above a certain threshold. All the tests were done without knowing the specific details of the experiment.

### 2.6 Statistical analysis

We used Prism 5.0 software to analyze the data. We used repeated measures two-way ANOVA to see if there were any differences in the behavioral tests. Then, we did a post-hoc analysis using a Bonferroni’s multiple comparison test to figure out the *p*-value for each of the experimental groups. We used one-way ANOVA to see if there were any differences in the selected date’s data analysis from the behavioral tests and immunohistochemistry. After that, we used Newman-Keuls multiple comparison test to look at more specific differences. The two-tailed Student’s t-test was performed to compare two groups. All the data from the experiments are shown as the average plus or minus the standard error of average. The *p* value was considered statistically significant at *p* < 0.05.

## 3 Results

### 3.1 Intrathecal progesterone administration during the induction phase enhances the development of mechanical allodynia in CCI mice

**FIGURE 1:**
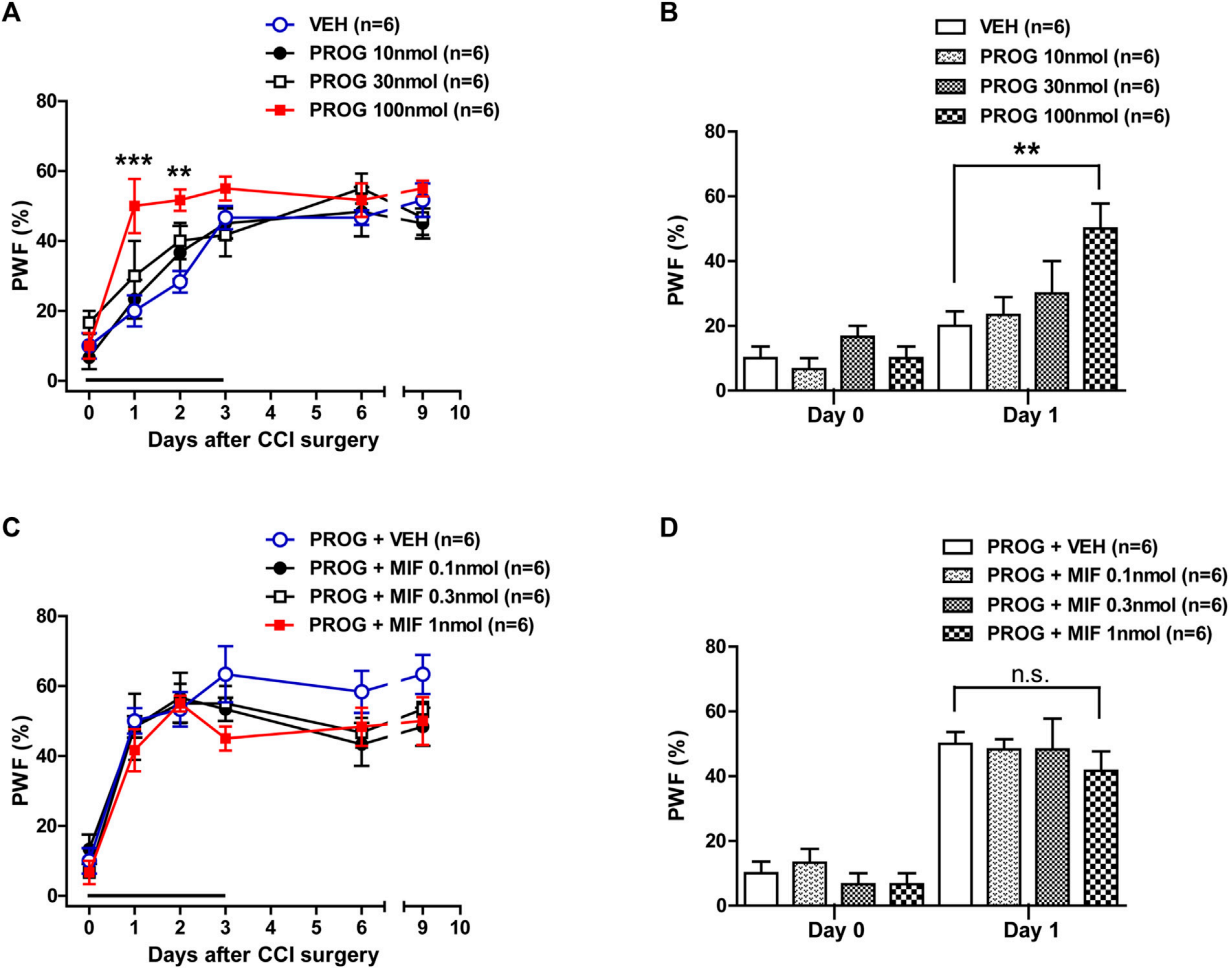
Effect of intrathecal administration of progesterone and the progesterone receptor antagonist, mifepristone on the chronic constriction injury (CCI)-induced mechanical allodynia during the induction phase of neuropathic pain (from days 0 to 3 post-surgery) in mice. **(A, B)** Repeated daily administration of progesterone (PROG; 10, 30 or 100 nmol) increased the paw withdrawal frequency (PWF, %) to innocuous mechanical stimuli in CCI mice **(A)**, and the data analysis at day 1 showed that progesterone significantly increases the CCI-induced development of mechanical allodynia **(B)**. **(C, D)** Conversely repeated daily administration of mifepristone (MIF; 0.1, 0.3 or 1 nmol) in combination with progesterone had no effect on the progesterone-induced increase in PWF **(C)**, and the data analysis at day 1 showed that there was no effect of mifepristone on the progesterone-induced increase in the CCI-induced mechanical allodynia **(D)**. *n* = 6 mice/group. **p < 0.01, ***p < 0.001 vs. VEH-treated CCI group. n.s., not significant.

In order to determine if progesterone modulates neuropathic pain following nerve injury, we examined the effect of intrathecal administration of progesterone (10, 30 or 100 nmol) on the CCI-induced mechanical allodynia during the induction phase of neuropathic pain. Sciatic nerve injury increased the paw withdrawal frequency (PWF, %) to innocuous mechanical stimuli in the hind paw from 3 days post-CCI surgery as compared with normal baseline values on day 0 (Figure 1A). Repeated intrathecal administration of progesterone during the induction phase of neuropathic pain (from days 0 to 3 post-surgery) induced an early increase in the PWF beginning at day 1 post-surgery as compared with the vehicle-treated CCI group (Figure 1A; **p < 0.01, ***p < 0.001 vs. VEH-treated CCI group; Group: F (3,120) = 7.032, *p* = 0.0002; Time: F (5,120) = 36.80, *p* < 0.0001; Interaction: F (15,120) = 1.433, *p* = 0.1427). In addition, the data analysis at day 1 showed a significant facilitatory effect of progesterone (100 nmol) on the development of mechanical allodynia in neuropathic mice (Figure 1B; **p < 0.01 vs. VEH-treated CCI group).

Next, we examined the effect of progesterone receptor antagonism in the progesterone-induced increase in mechanical allodynia by intrathecal administration of the progesterone receptor antagonist, mifepristone, which was co-administrated with progesterone. Repeated daily administration of mifepristone (0.1, 0.3 or 1 nmol in combination with progesterone) from days 0–3 post-surgery had no effect on the progesterone-induced enhancement of CCI associated mechanical allodynia (Figure 1C; Group: F (3,120) = 2.844, *p* = 0.0406; Time: F (5,120) = 45.63, *p* < 0.0001; Interaction: F (15,120) = 0.6622, *p* = 0.8165). The data analysis at day 1 showed that co-administration of mifepristone together with progesterone did not affect the facilitatory effect of progesterone alone on the CCI-induced increase in PWF (Figure 1D).

### 3.2 Progesterone-induced enhancement of mechanical allodynia is mediated by P450c17 activation in CCI mice

**FIGURE 2:**
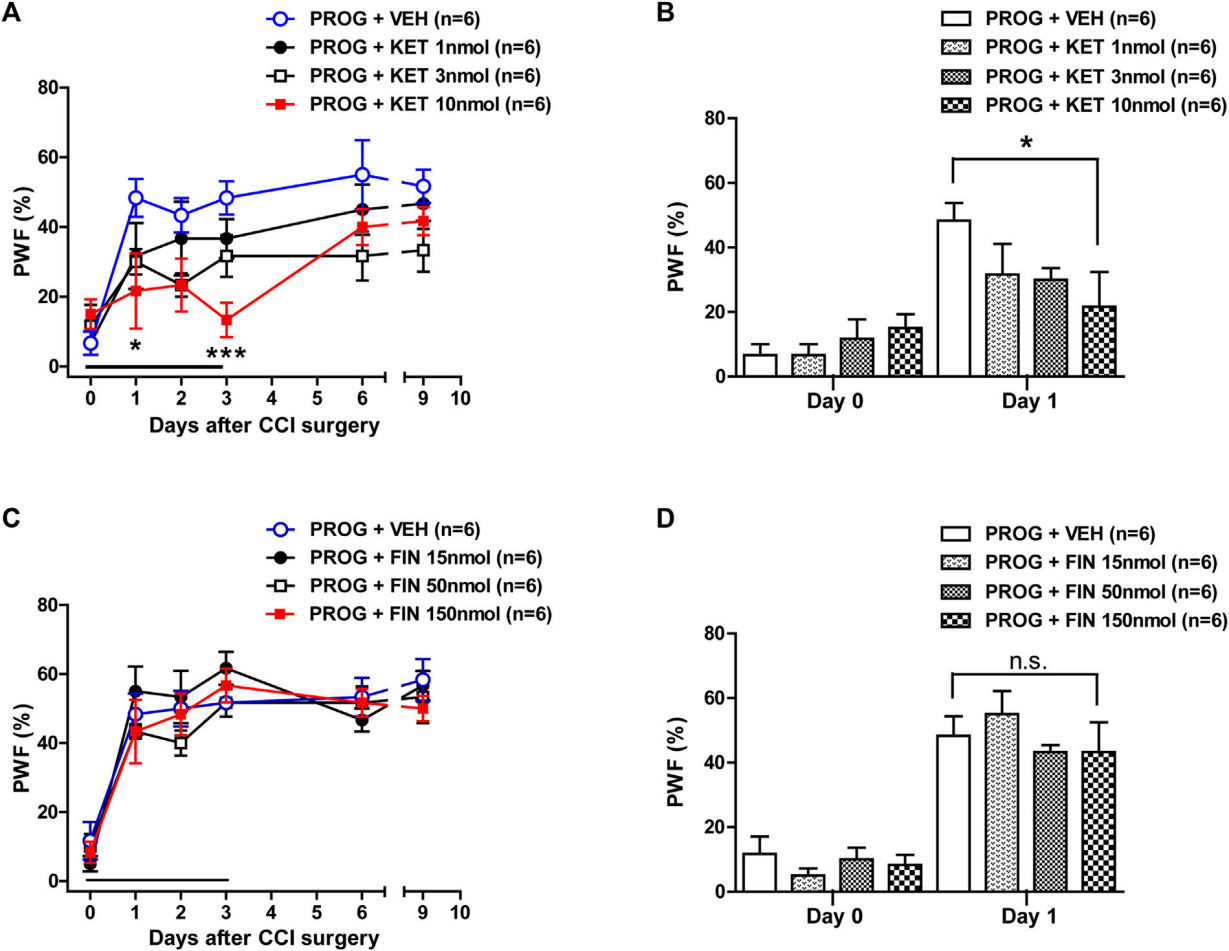
Effect of intrathecal co-administration of the P450c17 inhibitor, ketoconazole or the 5α-reductase inhibitor, finasteride in combination with progesterone during the induction phase of neuropathic pain (from days 0 to 3 post-surgery) on the chronic constriction injury (CCI)-induced mechanical allodynia in mice. **(A, B)** Repeated daily co-administration of ketoconazole (KET; 1, 3 or 10 nmol) together with progesterone (PROG; 100 nmol) reduced the paw withdrawal frequency (PWF, %) that was increased by administration of progesterone alone in CCI mice **(A)**, and the data analysis at day 1 showed a significant inhibitory effect of ketoconazole on progesterone-induced increase in PWF **(B)**. **(C, D)** Repeated daily co-administration of finasteride (FIN; 15, 50 or 150 nmol) together with progesterone (PROG; 100 nmol) had no effect on the PWF (%) that was increased by administration of progesterone alone in CCI mice **(C)**, and the data analysis at day 1 showed that there was no effect of finasteride on the progesterone-induced increase in PWF **(D)**. *n* = 6 mice/group. *p < 0.05, ***p < 0.001 vs. PROG+VEH-treated CCI group. n.s., not significant.

To determine whether spinal P450c17 or 5α-reductase modulates the progesterone-induced increase in mechanical allodynia, we examined the effect of intrathecal administration of the P450c17 inhibitor, ketoconazole or the 5α-reductase inhibitor, finasteride, which was co-administrated with progesterone. Repeated intrathecal administration of progesterone (100 nmol) from days 0 to 3 post-surgery induced an early increase in the paw withdrawal frequency (PWF, %) to innocuous mechanical stimuli (mechanical allodynia) in the hind paw (Figure 2A). Administration of ketoconazole (1, 3 or 10 nmol in combination with progesterone) from days 0 to 3 post-surgery inhibited this progesterone-induced enhancement of CCI associated mechanical allodynia (Figure 2A; *p < 0.05, ***p < 0.001 vs. PROG+VEH-treated CCI group; Group: F (3,120) = 8.474, *p* < 0.0001; Time: F (5,120) = 14.48, *p* < 0.0001; Interaction: F (15,120) = 1.319, *p* = 0.2013). In addition, the data analysis at day 1 showed a significant inhibitory effect of ketoconazole (10 nmol) on the progesterone-induced facilitation of mechanical allodynia in neuropathic mice (Figure 2B; *p < 0.01 vs. PROG+VEH-treated CCI group).

By contrast, repeated intrathecal administration of finasteride (15, 50 or 150 nmol in combination with progesterone) from days 0 to 3 post-surgery had no effect on the progesterone-induced enhancement of CCI associated mechanical allodynia (Figure 2C; Group: F (3,120) = 1.187, *p* = 0.3180; Time: F (5,120) = 45.85, *p* < 0.0001; Interaction: F (15,120) = 0.5872, *p* = 0.8798). The data analysis at day 1 showed that co-administration of finasteride (150 nmol) together with progesterone did not affect the facilitatory effect of progesterone alone on the CCI-induced increase in PWF (Figure 2D).

### 3.3 Intrathecal progesterone administration during the induction phase increases spinal astrocyte activation and this increase is mediated by P450C17 in CCI mice

**FIGURE 3:**
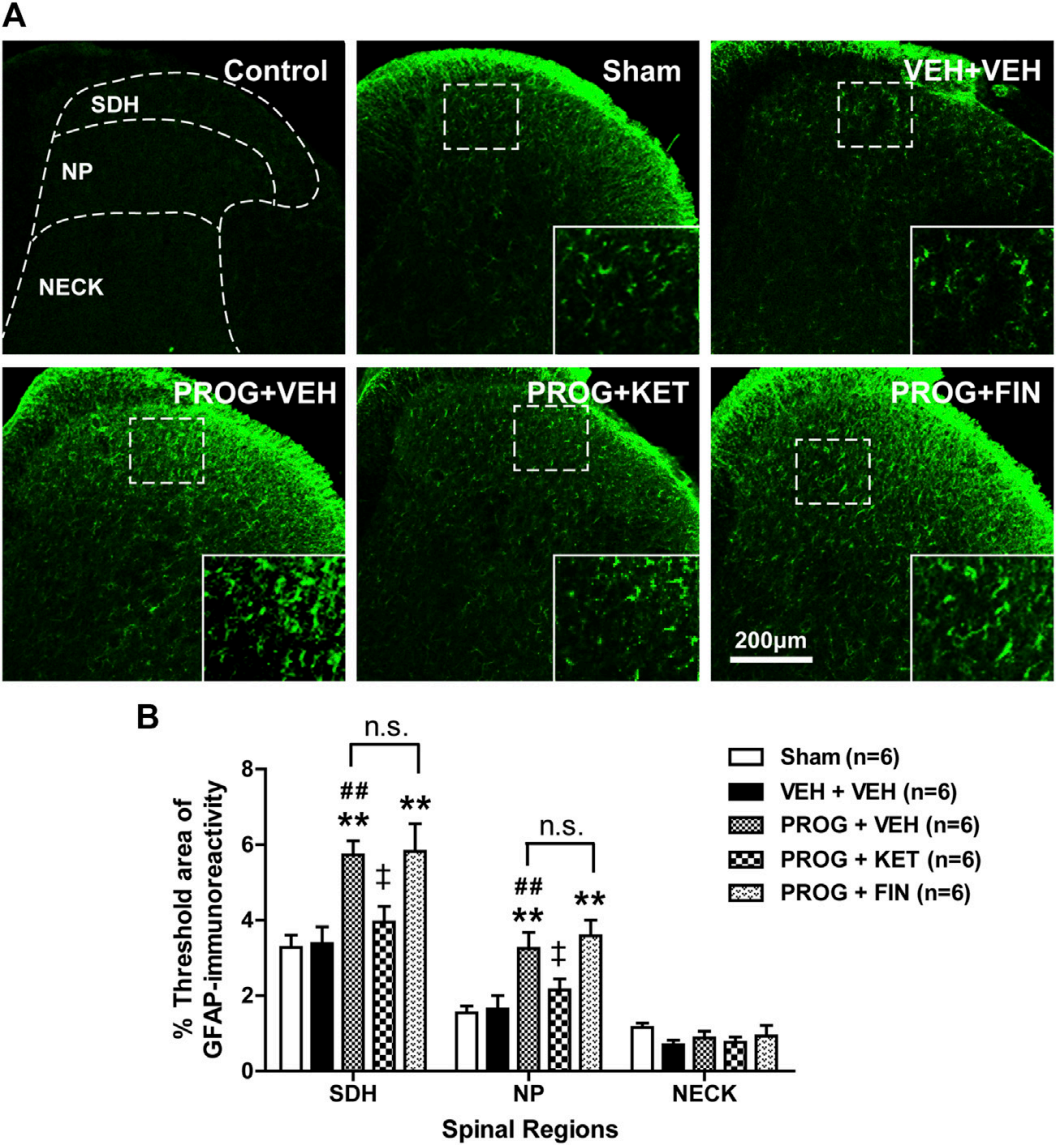
Effect of intrathecal co-administration of the P450c17 inhibitor, ketoconazole or the 5α-reductase inhibitor, finasteride in combination with progesterone during the induction phase of neuropathic pain (from days 0 to 1 post-surgery) on the chronic constriction injury (CCI)-induced spinal glial fibrillary acidic protein (GFAP) expression in mice. **(A, B)** Representative immunohistochemistry images **(A)** and a graph **(B)** showed that administration of progesterone increased the immunofluorescence of GFAP in the superficial dorsal horn (SDH, lamina I-II) and nucleus proprius (NP, lamina III-IV) regions, but not in neck region (NECK, lamina V-VI) of mice at day 1 post-surgery. Co-administration of ketoconazole (KET; 10 nmol) together with progesterone (PROG; 100 nmol) reduced the progesterone-induced increased GFAP-immunoreactivity in the spinal cord at day 1 post-surgery in CCI mice, while co-administration of finasteride (FIN; 150 nmol) together with progesterone (PROG; 100 nmol) had no effect on the progesterone-induced increased GFAP-immunoreactivity in the spinal cord. As a negative control, immunostaining was performed without primary antibody. Scale bar = 200 μm. *n* = 6 mice/group. **p < 0.01 vs. Sham; ##*p* < 0.01 vs. VEH+VEH-treated CCI group; ‡*p* < 0.05 vs. PROG+VEH-treated CCI group. n.s., not significant.

To determine whether progesterone modulates the pathological activation of spinal astrocytes during the induction phase of neuropathic pain, we examined the effect of intrathecal administration of progesterone (100 nmol) on the expression of GFAP, a specific marker for astrocyte reactivity, in the lumbar spinal cord dorsal horn of CCI mice. Immunohistochemical analysis showed that GFAP-immunoreactivity did not change in the lumbar spinal cord of CCI mice at day 1 post-surgery as compared with sham group (Figures 3A, B). Repeated intrathecal administration of progesterone (100 nmol) significantly increased GFAP-immunoreactivity in the superficial dorsal horn (SDH, laminae I-II) and nucleus proprius (NP, laminae III-IV) regions of the lumbar spinal cord dorsal horns at day 1 post-surgery as compared with sham and vehicle-treated CCI groups (Figures 3A, B; **p < 0.01 vs. Sham group; ##*p* < 0.01 vs. VEH-treated CCI group).

Repeated intrathecal administration of ketoconazole (10 nmol in combination with progesterone) from days 0–1 post-surgery inhibited the progesterone-induced enhancement of GFAP-immunoreactivity in the superficial dorsal horn (SDH, laminae I-II) and nucleus proprius (NP, laminae III-IV) regions of the lumbar spinal cord dorsal horns at day 1 post-surgery as compared with vehicle-treated CCI group (Figures 3A, B; †*p* < 0.05 vs. PROG+VEH-treated CCI group). By contrast, intrathecal administration of finasteride (150 nmol in combination with progesterone) from days 0 to 1 post-surgery had no effect on the progesterone-induced enhancement of GFAP-immunoreactivity during the induction phase of CCI-induced neuropathy (Figures 3A, B; **p < 0.01 vs. VEH-treated CCI group).

### 3.4 Sciatic nerve injury-induced changes in the phospho-serine levels of P450c17 in the lumbar spinal cord dorsal horn of CCI mice

**FIGURE 4:**
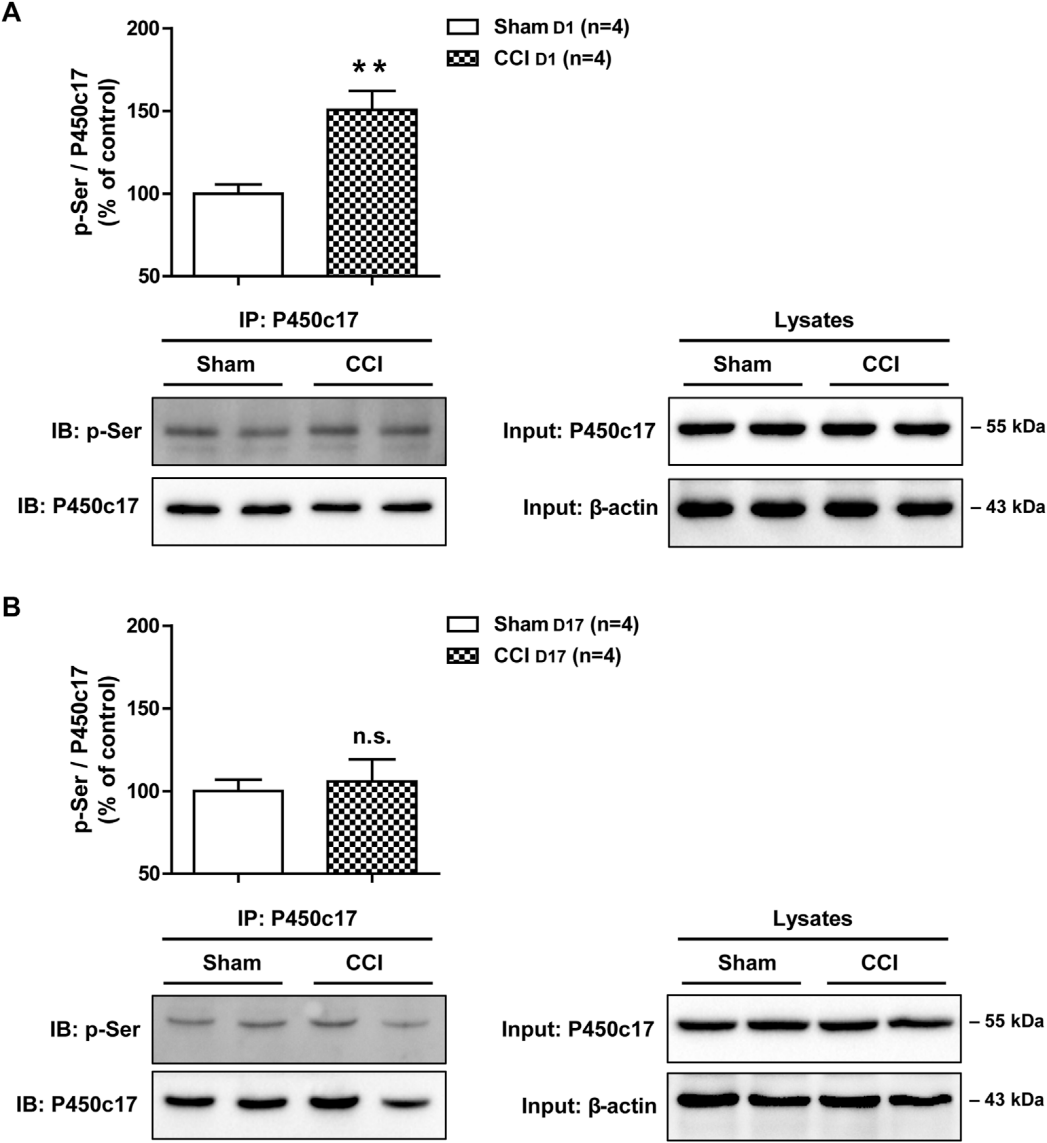
Effect of chronic constriction injury (CCI) of the sciatic nerve on the phospho-serine levels of P450c17 in the spinal cord dorsal horn of CCI mice. **(A, B)** A co-immunoprecipitation method was used to determine the phospho-serine levels of P450c17 at post-operative day 1 **(A)** or day 17 **(B)** in the spinal cord. Homogenates were immunoprecipitated with anti-P450c17 antibody, then blotted (IB) with anti-phosphoserine (p-Ser) antibody. Sciatic nerve injury increased the phospho-serine levels of P450c17 at day 1 post-surgery, but not at day 17 post-surgery. Input, 20 μg of tissue lysates from each group. *n* = 4 mice/group. **p < 0.01 vs. Sham. n.s., not significant.

Since progesterone is catalyzed by cytochrome P450c17, it was important to determine the duration of the actions of intrathecal progesterone on the activity of P450c17. Since phosphorylation of P450c17 on serine residues increases the activity of this enzyme (Tee et al., 2008), we examined the CCI-induced changes in the phospho-serine levels of P450c17 using co-immunoprecipitation experiments followed by Western blot analysis. Sciatic nerve injury induced a significant increase in the phospho-serine levels of P450c17 in the lumbar spinal cord dorsal horn at day 1 post-surgery when compared to sham surgery mice (Figure 4A; **p < 0.01 vs. Sham group; T (6) = 3.939, *p* = 0.0076). By contrast, there was no difference in the phospho-serine levels of P450c17 between sham and CCI groups at day 17 post-surgery (Figure 4B; T (6) = 0.3950, *p* = 0.7065).

### 3.5 Intrathecal progesterone administration during the maintenance phase suppresses the developed mechanical allodynia in CCI mice

**FIGURE 5:**
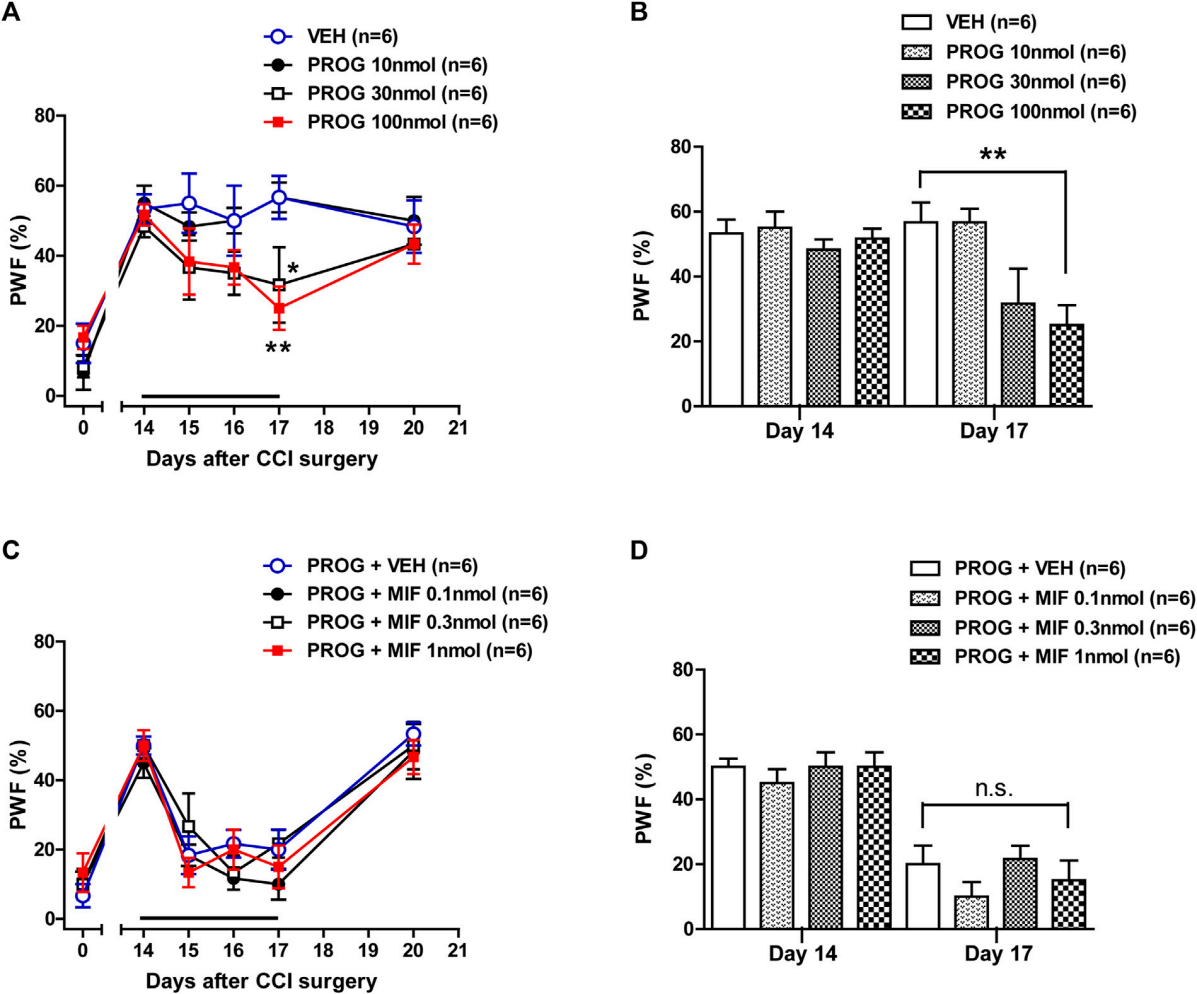
Effect of intrathecal administration of progesterone and the progesterone receptor antagonist, mifepristone on the chronic constriction injury (CCI)-induced mechanical allodynia during the maintenance phase of neuropathic pain (from days 14 to 17 post-surgery) in mice. **(A, B)** Repeated daily administration of progesterone (PROG; 10, 30 or 100 nmol) decreased the paw withdrawal frequency (PWF, %) to innocuous mechanical stimuli in CCI mice **(A)**, and the data analysis at day 17 showed that progesterone significantly suppresses the CCI-induced developed mechanical allodynia **(B)**. **(C, D)** Conversely repeated daily administration of mifepristone (MIF; 0.1, 0.3 or 1 nmol) in combination with progesterone had no effect on the progesterone-induced increase in PWF **(C)**, and the data analysis at day 17 showed that there was no effect of mifepristone on the progesterone-induced increase in the CCI-induced mechanical allodynia **(D)**. *n* = 6 mice/group. *p < 0.05, **p < 0.01 vs. VEH-treated CCI group. n.s., not significant.

Intrathecal administration of progesterone during the maintenance phase of chronic pain (post-operative days 14–17) significantly attenuated the CCI-induced increase in PWF (Figure 5A; *p < 0.05, **p < 0.01 vs. VEH-treated CCI group; Group: F (3,120) = 6.067, *p* = 0.0007; Time: F (5,120) = 20.66, *p* < 0.0001; Interaction: F (15,120) = 1.114, *p* = 0.3516). The data analysis at day 17 showed a significant analgesic effect of progesterone on the developed mechanical allodynia in neuropathic mice (Figure 5B; **p < 0.01 vs. VEH-treated CCI group). I.t. administration of mifepristone (0.1, 0.3 or 1 nmol) in combination with progesterone on post-operative days 14–17 had no effect on the progesterone-induced inhibition of CCI-induced mechanical allodynia (Figure 5C; Group: F (3,120) = 1.109, *p* = 0.3482; Time: F (5,120) = 48.07, *p* < 0.0001; Interaction: F (15,120) = 0.5875, *p* = 0.8797). The data analysis at day 17 showed that co-administration of mifepristone together with progesterone did not affect the analgesic effect of progesterone alone on the CCI-induced increase in PWF (Figure 5D).

### 3.6 Progesterone-induced analgesic effect on the developed mechanical allodynia is mediated by 5α-reductase activation in CCI mice

**FIGURE 6:**
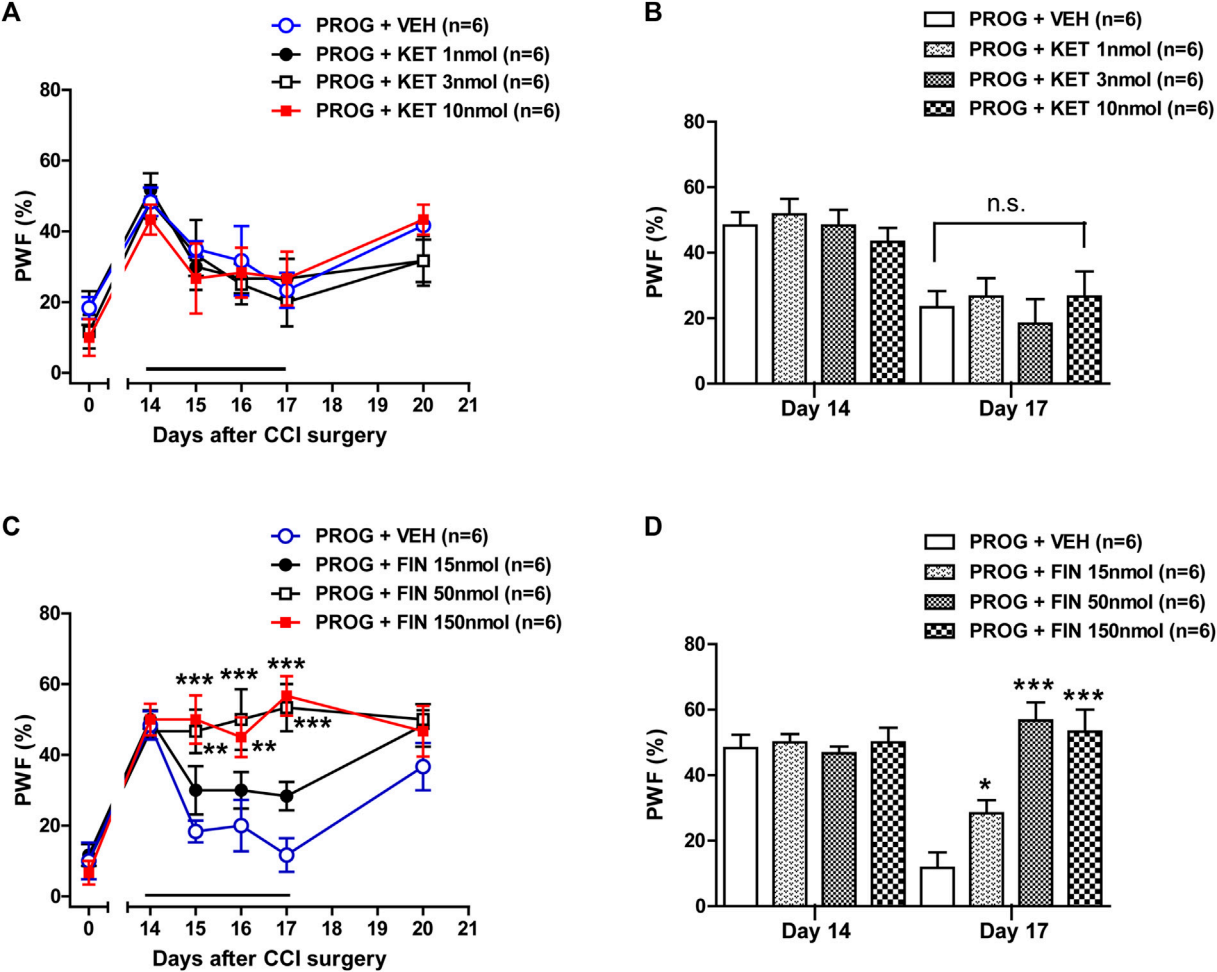
Effect of intrathecal co-administration of the P450c17 inhibitor, ketoconazole or the 5α-reductase inhibitor, finasteride in combination with progesterone during the maintenance phase of neuropathic pain (from days 14 to 17 post-surgery) on the chronic constriction injury (CCI)-induced mechanical allodynia in mice. **(A, B)** Repeated daily co-administration of ketoconazole (KET; 1, 3 or 10 nmol) together with progesterone (PROG; 100 nmol) had no effect on the paw withdrawal frequency (PWF, %) that was decreased by administration of progesterone alone in CCI mice **(A)**, and the data analysis at day 17 showed that there was no effect of ketoconazole on progesterone-induced decrease in PWF **(B)**. **(C, D)** Repeated daily co-administration of finasteride (FIN; 15, 50 or 150 nmol) together with progesterone (PROG; 100 nmol) restored the PWF (%) that was decreased by administration of progesterone alone in CCI mice **(C)**, and the data analysis at day 17 showed the effect of finasteride on the progesterone-induced decrease in PWF **(D)**. *n* = 6 mice/group. *p < 0.05, **p < 0.01, ***p < 0.001 vs. PROG+VEH-treated CCI group. n.s., not significant.

Intrathecal administration of ketoconazole (1, 3 or 10 nmol) (Figure 6A; Group: F (3,120) = 0.6910, *p* = 0.5593; Time: F (5,120) = 14.99, *p* < 0.0001; Interaction: F (15,120) = 0.4552, *p* = 0.9578) in combination with progesterone on post-operative days 14–17 had no effect on the progesterone-induced inhibition of CCI-induced mechanical allodynia. The data analysis at day 17 showed that co-administration of ketoconazole together with progesterone did not affect the analgesic effect of progesterone alone on the CCI-induced increase in PWF (Figure 6B). By contrast, administration of finasteride (15, 50 or 150 nmol) restored the progesterone-induced inhibition of CCI associated mechanical allodynia (Figure 6C; **p < 0.01, ***p < 0.001 vs. PROG+VEH-treated CCI group; Group: F (3,120) = 16.22, *p* < 0.0001; Time: F (5,120) = 26.09, *p* < 0.0001; Interaction: F (15,120) = 2.988, *p* = 0.0004). The data analysis at day 17 showed a significant inhibitory effect of finasteride on the progesterone-induced inhibition of mechanical allodynia in neuropathic mice (Figure 6D; *p < 0.05, ***p < 0.001 vs. PROG+VEH-treated CCI group).

### 3.7 Intrathecal progesterone administration during the maintenance phase inhibits spinal astrocyte activation and this inhibition is mediated by 5α-reductase in CCI mice

**FIGURE 7:**
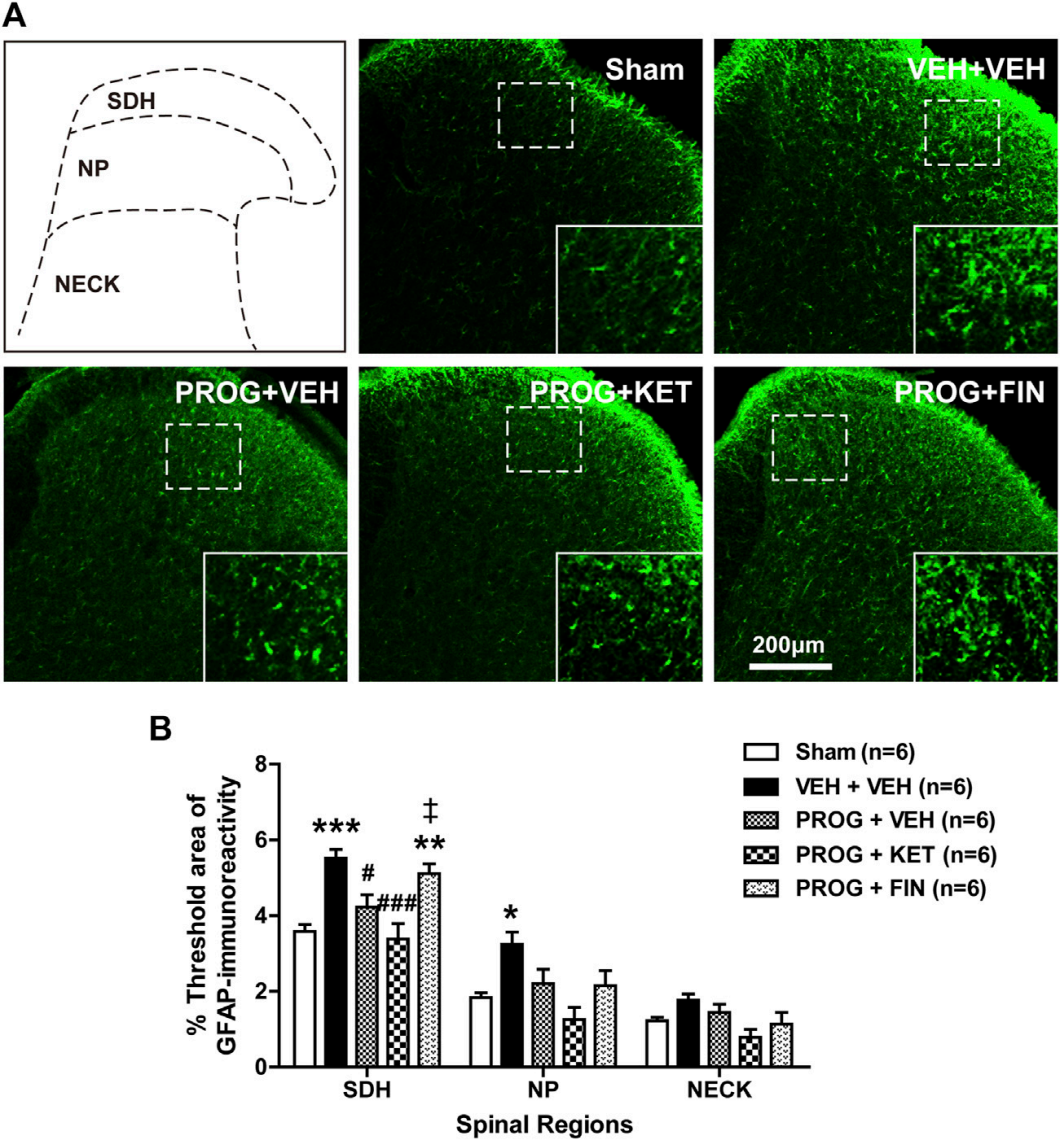
Effect of intrathecal co-administration of the P450c17 inhibitor, ketoconazole or the 5α-reductase inhibitor, finasteride in combination with progesterone during the maintenance phase of neuropathic pain (from days 14 to 17 post-surgery) on the chronic constriction injury (CCI)-induced spinal glial fibrillary acidic protein (GFAP) expression in mice. **(A, B)** Representative immunohistochemistry images **(A)** and a graph **(B)** showed that administration of progesterone decreased the immunofluorescence of GFAP in the superficial dorsal horn (SDH, lamina I-II) of mice at day 17 post-surgery. Co-administration of ketoconazole (KET; 10 nmol) together with progesterone (PROG; 100 nmol) had no effect on the progesterone-induced decreased GFAP-immunoreactivity in the spinal cord at day 17 post-surgery in CCI mice, while co-administration of finasteride (FIN; 150 nmol) together with progesterone (PROG; 100 nmol) restored the progesterone-induced decreased GFAP-immunoreactivity in the spinal cord. Scale bar = 200 μm. *n* = 6 mice/group. *p < 0.05, **p < 0.01, ***p < 0.001 vs. Sham; #*p* < 0.05, ###*p* < 0.001 vs. VEH+VEH-treated CCI group; ‡*p* < 0.05 vs. PROG+VEH-treated CCI group. n.s., not significant.

In contrast to the induction phase of chronic pain, during the maintenance phase i.t. administration of progesterone (100 nmol) on post-operative days 14-17 significantly reduced the CCI-induced increased GFAP-immunofluorescence in the SDH region at day 17 post-surgery (Figures 7A, B; *p < 0.05, ***p < 0.001 vs. Sham group; #*p* < 0.05 vs. VEH+VEH-treated CCI group). To determine the potential role of P450c17 or 5α-reductase in the progesterone-induced inhibition of astrocyte activation, we examined the effect of ketoconazole, a P450c17 inhibitor, or finasteride, a 5α-reductase inhibitor, which was intrathecally co-administrated with progesterone on post-operative days 14-17. In contrast to the induction phase of chronic pain, administration of ketoconazole (10 nmol) had no effect on the progesterone-induced suppression of GFAP-immunofluorescence at day 17 post-CCI surgery, while administration of finasteride significantly restored the progesterone-induced inhibition of GFAP-immunofluorescence in the SDH (laminae I-II) region (Figures 7A, B; **p < 0.01 vs. Sham group; ###*p* < 0.001 vs. VEH+VEH-treated CCI group; ‡*p* < 0.05 vs. PROG+VEH-treated CCI group).

## 4 Discussion

**FIGURE 8:**
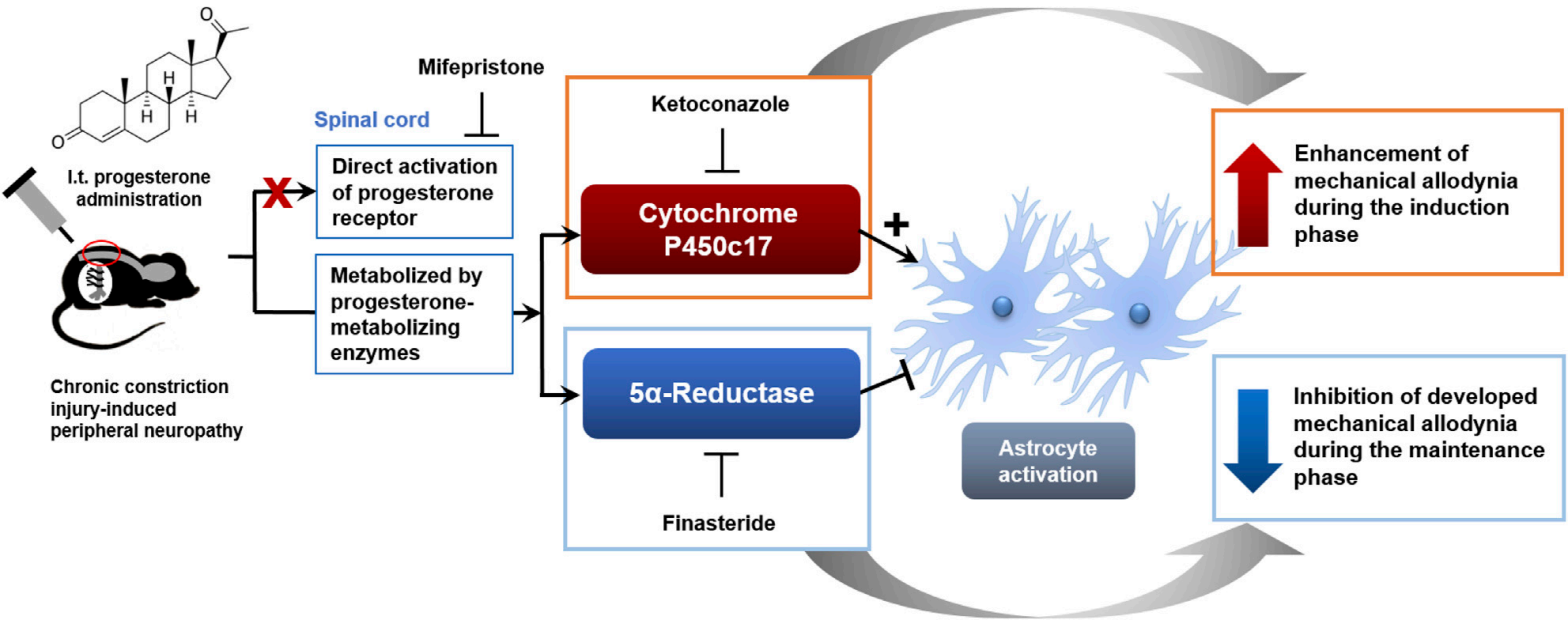
Schematic diagram summarizing the proposed mechanisms underlying phase-specific differential regulation of mechanical allodynia in a murine model of neuropathic pain by progesterone. Administration of progesterone during the early phase of neuropathic pain facilitates the development of mechanical allodynia, as well as, the pathological activation of spinal astrocytes and that these actions of progesterone are mediated by the activation of P450c17. Administration of progesterone during the late phase of neuropathic pain inhibits the developed mechanical allodynia and the activation of spinal astrocytes and that these actions of progesterone are mediated by the activation of 5α-reductase.

There are three important novel findings resulting from this study that support our original hypothesis. First, repeated i.t. administration of progesterone from days 0 to 3 post-surgery significantly facilitated mechanical allodynia development and also increased the pathological activation of astrocytes in the lumbar spinal dorsal horn of CCI mice. These progesterone-induced changes were suppressed by inhibition of P450c17 with ketoconazole administration, but not by inhibition of 5α-reductase with finasteride administration. Secondly, sciatic nerve injury increases the phospho-serine levels of P450c17 at post-operative day 1, while the phospho-serine levels of P450c17 was not altered at post-operative day 17 in CCI mice. Finally, repeated i.t. administration of progesterone from days 14 to 17 post-surgery inhibited both the established mechanical allodynia and spinal astrocyte activation in CCI mice. These changes were suppressed by inhibition of 5α-reductase, but not by inhibition of P450c17. Collectively, these results demonstrate that i.t. administration of progesterone during the maintenance phase of chronic pain has an analgesic effect on the mechanical allodynia associated with the developing neuropathy and also inhibits spinal astrocyte activation via the action of 5α-reductase. However, administration of progesterone during the early phase of chronic pain facilitates mechanical allodynia development, as well as, the pathological activation of astrocytes and that these actions of progesterone result from the increased activity of P450c17 during the early induction period following peripheral nerve injury (Figure 8).

Progesterone can be converted to other steroids such as dihydroprogesterone via 5α-reductase (Arbo et al., 2016). Peng et al. showed that administration of progesterone produces gamma-aminobutyric acid type A (GABA_A_) receptors-dependent inhibition of the induction of spinal reflex potentiation and that this change is blocked by inhibition of 5α-reductase, but not by antagonism of progesterone receptor (Peng et al., 2009). These results suggest that progesterone-mediated effect on neural inhibition is regulated by neurosteroid metabolites rather than progesterone receptor activation. Allopregnanolone, a reduced metabolite of progesterone, is a positive allosteric modulator of the GABA_A_ receptors and intraplantar administration of allopregnanolone alleviates thermal and mechanical hyperalgesia in a rat model of neuropathic pain via stimulation of peripheral GABA_A_ receptors (Belelli and Lambert, 2005; Pathirathna et al., 2005; Patte-Mensah et al., 2014; Arbo et al., 2016). Thus, allopregnanolone synthetic pathway initiated by 5α-reductase from progesterone could elicit analgesic actions on nociception. Conversely our results showed that inhibition of 5α-reductase with finasteride has no effect on the progesterone-induced enhancement of mechanical allodynia during the induction phase of neuropathic pain. These results raise the possibility that progesterone-mediated modulation of nociception during the early induction period following peripheral neuropathy occurs via another mechanism, which is different from allopregnanolone synthetic pathway and progesterone receptor activation.

On the other hand, the activation of cytochrome P450c17 can convert progesterone into another neurosteroid, androstenedione (Do Rego et al., 2009). Androstenedione can be metabolized to testosterone and further converted to 17β-estradiol by the action of the cytochrome P450 aromatase (Arbo et al., 2016). This multistep process triggered by P450c17 from progesterone has been suggested to elicit allodynic actions on nociception. Inhibition of spinal aromatase with letrozole significantly inhibits formalin-induced nociceptive behaviors and Fos expression in the spinal cord dorsal horn (Choi et al., 2018). In addition, administration of letrozole reduces tumor-induced hyperalgesia in female mice suggesting that spinal estrogen contributes to the development of bone tumor-induced hyperalgesia (Smeester et al., 2016). This is consistent with other studies in the spinal cord that have demonstrated that 17β-estradiol increases spinal processing of visceral nociception by increasing both the expression and the phosphorylation of N-methyl-D-aspartate (NMDA) receptor GluN1 subunit contributing to an increase in NMDA receptor activity (Tang et al., 2008). In a previous study from our laboratories, we demonstrated that inhibition of spinal P450c17 during the induction phase of neuropathic pain significantly suppresses the development of CCI-induced mechanical allodynia showing that spinal P450c17 plays an important role in the development of chronic pain following peripheral nerve injury (Choi et al., 2019c). In the present study, the phospho-serine levels of P450c17 were increased on post-operative day 1, during the early phase of neuropathic pain development following sciatic nerve injury. Since phosphorylation of P450c17 on serine residues increases 17,20-lyase activity (Tee et al., 2008; Tee and Miller, 2013), it is possible that intrathecally administrated progesterone is rapidly metabolized during the induction phase of neuropathic pain through the initial activation of the P450c17 enzyme and that this process may elicit the allodynic action of progesterone treatment on the development of neuropathic pain.

There are several studies in the literature showing the possible modulatory mechanisms related to neurosteroidogenesis during the induction phase of pain. Peripheral nerve injury causes a significant increase in the expression of phosphorylated mitogen-activated protein kinases, such as p38 and extracellular signal regulated kinase 1/2, in the spinal cord (Tsuda et al., 2004; Díaz et al., 2019). Especially, p38α is the principal kinase that phosphorylates P450c17 and increases the maximum velocity of the 17,20-lyase reaction (Tee and Miller, 2013). In a previous study, we demonstrated that p38 activation is increased during the induction phase of nerve injury and plays an important role in the development of mechanical allodynia in a rat model of neuropathic pain (Moon et al., 2013). Thus, early activation of p38 may increase neurosteroidogenesis via phosphorylation-mediated activation of P450c17. In addition, the activity of spinal aromatase is increased by the activation of sigma-1 receptors in a mouse model of inflammatory pain (Choi et al., 2018). It has been suggested that spinal sigma-1 receptors play an important role during the induction phase of neuropathic pain (Roh et al., 2008; Choi et al., 2016), thus, sigma-1 receptor-induced activation of aromatase may modulate the nerve injury-induced early neurosteroidogenesis. Furthermore, Patte-Mensah and colleagues suggested that substance P inhibits progesterone conversion into allopregnanolone in spinal sensory circuit (Patte-Mensah et al., 2005). Since substance P plays a role in the early phase of nociceptive signal transmission (Greco et al., 2008; Sahbaie et al., 2009), it is possible that nerve injury-induced increased release of substance P inhibits the conversion of progesterone into allopregnanolone, which is initiated by 5α-reductase, resulting in a reduction of GABAergic transmission during the early phase of peripheral neuropathy. Since the activity of neurosteroidogenic enzymes may be modulated differently during the early phase of chronic pain, steroid-based therapies should be performed with consideration of the changes in the activity of diverse neurosteroidogenic enzymes under pathophysiological condition.

Abnormal activation of astrocytes occurs in the spinal cord following nerve injury and these seem to play an important role in the pathological changes that occur in second order neurons and other glial cells in the nervous system (Watkins et al., 2001; Moon et al., 2015; Choi et al., 2016). Activated astrocytes synthesize and release diverse proinflammatory cytokines (interleukin-1β, interleukin-6 and tumor necrosis factor-α) and gliotransmitters (D-serine, ATP and glutamate) and these signaling molecules have been shown to mediate nociceptive signal transmission in the spinal cord in different animal models of inflammatory and neuropathic pain (Watkins et al., 2001; Raghavendra et al., 2004; De Leo et al., 2006; Moon et al., 2015). In the present study, intrathecal administration of progesterone modulated the activation of astrocytes located in both the SDH (laminae I-II) and NP (laminae III-IV) regions of the lumbar spinal cord dorsal horns in CCI mice. Progesterone-induced modulation of astrocyte activation was significantly attenuated by inhibition of P450c17, but not 5α-reductase, and these effects were correlated with the CCI-induced changes in mechanical allodynia. Since it has been suggested that primary afferent neurons convey nociceptive signaling to second order neurons and glial cells located mainly in the SDH and NP regions of the spinal cord dorsal horn (Todd, 2010), progesterone- and P450c17-induced modulation of astrocytes in these regions could certainly affect spinal nociceptive signal transmission following peripheral nerve injury. In future studies, we plan to investigate the related receptors and signal transduction pathways, which play a major role in the progesterone-induced modulation of nociception and astrocyte activation.

## 5 Conclusion

The present study demonstrates that sciatic nerve injury increases the activity of P450c17 (a metabolic enzyme of progesterone, as evidenced by a significant increase in phospho-serine levels of P450c17) in the spinal cord of CCI mice during the early phase of neuropathic pain. This increase results in an enhanced allodynic effect of progesterone administration during the induction phase of neuropathic pain. Furthermore, our results show that progesterone-induced early activation of spinal astrocytes is mediated by the activation of P450c17, but not 5α-reductase. Thus, the actions of progesterone are different depending on the activation of the metabolic enzymes, P450c17 and 5α-reductase, and this finding may help explain the discrepancies in previous studies regarding the analgesic or nociceptive effects of this important steroid. This study provides new insights into a potential role of P450c17 in progesterone-induced modulation of nociception and further suggests that in order to develop a more efficacious progesterone therapy for neuropathic pain control, the activity of P450c17 needs to be considered.

## Data Availability

The raw data supporting the conclusion of this article will be made available by the authors, without undue reservation.
